# Association Between Platelet-to-Lymphocyte Ratio and Preterm Necrotizing Enterocolitis

**DOI:** 10.3389/fped.2021.686880

**Published:** 2021-11-05

**Authors:** Juan Zheng, Hua Wang

**Affiliations:** ^1^Department of Pediatrics, West China Second University Hospital, Sichuan University, Chengdu, China; ^2^Key Laboratory of Birth Defects and Related Diseases of Women and Children, Ministry of Education, Sichuan University, Chengdu, China; ^3^Department of Pediatrics, Zigong Fourth People's Hospital, Zigong, China

**Keywords:** NEC, PLR, association, preterm neonates, prediction, generalized additive models

## Abstract

**Introduction:** Necrotizing enterocolitis (NEC) is a fatal condition for very-low-birth-weight infants. Necrotizing enterocolitis is a multi-factor phenomenon that results in intestinal mucosal damage and leads to intestinal necrosis. However, sensitive laboratory indicators for NEC are lacking, making early diagnosis difficult. This study aimed to explore the relationship between the platelet-to-lymphocyte ratio (PLR) and NEC in preterm neonates to enable an earlier diagnosis of the condition.

**Methods:** This was a retrospective case–control study of preterm neonates diagnosed with NEC between January 2018 and December 2019 in the West China Second University Hospital. Controls were selected from preterm neonatal intensive care unit (NICU) graduates, and they were matched for gestation and year of birth to the preterms diagnosed without NEC. In total, 93 and 107 infants were included in the NEC and control groups, respectively. Empowerstats analysis was used to identify the association between PLR and preterm NEC.

**Results:** The NEC group had significantly higher PLR levels than the control group. PLR > 100 within 1 week before NEC diagnosis was a risk factor for NEC. There was a positive connection between PLR and preterm NEC. A PLR of >100 was determined as the optimal cutoff for predicting preterm NEC, with patients with PLR >100 having a higher risk of NEC [odds ratio (OR): 18.82 (95% confidence interval (CI): 2.93–120.98), *p* = 0.002].

**Conclusions:** A PLR of >100 within 1 week after clinical abnormalities is associated with a high risk of NEC in preterm neonates.

## Introduction

Necrotizing enterocolitis (NEC) is one of the most common gastrointestinal emergencies in newborn infants (1). It is a neonatal inflammatory bowel disease characterized by hemorrhagic and necrotizing inflammation of the various layers of the intestinal wall. Premature infants with a birth weight of <750 g have a 42% mortality rate due to NEC, while those with a birth weight of 1,250–1,500 g have a lower mortality rate (2). In 1978, the Bell staging criteria, in which NEC is evaluated according to the development of inflammation, were established (3). In 1986, Walsh and Kliegman modified the staging criteria (4). The modified version has been used till date. The pathophysiological mechanism of NEC is yet to be fully elucidated (5, 6). Further, clinical manifestations at onset are usually non-specific, and conservative or surgical treatment cannot prevent the intramural inflammation. As such, emphasis is placed on prevention.

The platelet-to-lymphocyte ratio (PLR) has attracted increasing research attention as a new potential inflammatory marker. It has been found to predict the risk of cardiovascular disease and tumors and to be also associated with inflammatory diseases. In these conditions, a higher PLR is associated with poorer prognosis (7–11). However, to the best of our knowledge, there has been no research on the relationship between PLR and early NEC. Thus, this study aimed to explore the relationship between PLR and preterm NEC and to determine whether a high PLR is associated with a higher risk of NEC.

## Materials and Methods

### Study Design and Patients

**Figure 1:**
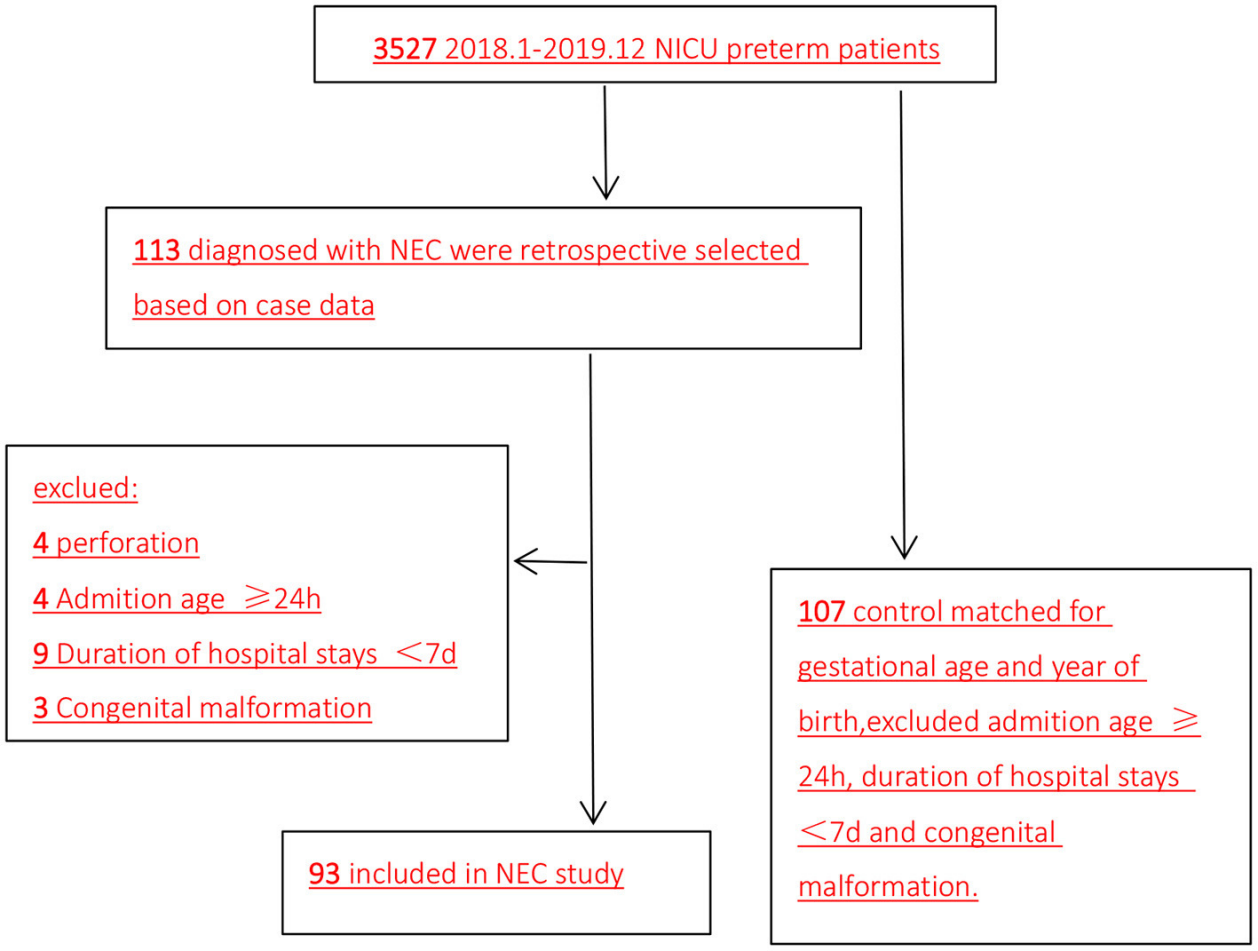
Flow diagram of patient selection (2018.1–2019.12). NICU, neonatal intensive care unit; NEC, necrotizing enterocolitis.

This was a retrospective case–control study of neonatal intensive care unit (NICU) preterm infants who developed NEC between January 2018 and December 2019. The patients were from the NICU of Sichuan University West China Second University Hospital. Among the 3,527 preterm infants, 133 infants were diagnosed with NEC. After excluding 20 infants due to perforation (*n* = 4), admission ≥24 h after birth (*n* = 4), admission <7 days (*n* = 9), and congenital malformation (*n* = 3), 93 preterm infants diagnosed with NEC were included in the NEC group. Meanwhile, the control group included 107 preterm infants without NEC matched for gestational age and year of birth (Figure 1). Infants who receive high-dose or exclusive breastfeeding within 14 days of birth have lower risk of NEC (12, 13). Thus, in our study, all infants were on mixed feeding, and there was no significant difference in milk selection and increase rate.

### Diagnosis of Necrotizing Enterocolitis

Necrotizing enterocolitis was diagnosed according to the Bell and Walsh grading criteria and revised consensus, including Stage I, Stage II, and Stage IIIA (3, 4).

### Calculation of the Platelet-to-Lymphocyte Ratio

Platelet-to-lymphocyte ratio was calculated as the mean ratio of platelet count to lymphocyte count in all blood tests within 1 week before NEC diagnosis.

### Statistical Analysis

**Figure 2:**
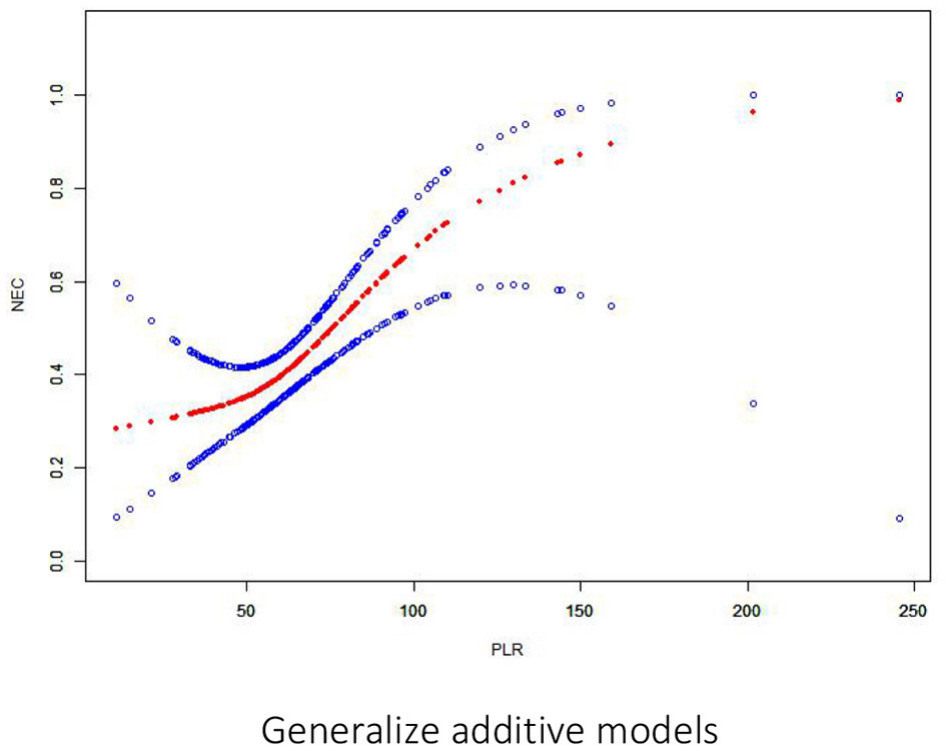
Association between PLR and NEC. There is a threshold and non-linear association between PLR and NEC (*p* < 0.05) in the generalized additive model (GAM). The smooth curve fit between variables is represented by a solid red line. The 95% confidence interval from the fit is represented by blue bands.

First, normally and non-normally distributed continuous variables were analyzed using the *t*-test and the Kruskal–Wallis rank sum test. Meanwhile, the χ^2^-test was used for categorical data to compare the data distribution of each covariate between the exposed and the non-exposed groups (Table 1). Next, generalized additive model curve analysis was done to evaluate the diagnostic capability of PLR for NEC (Figure 2), and univariate (Table 2) and multivariate logistic regression (Table 3) models were performed to examine whether PLR is associated with NEC. A *p* < 0.05 indicates a statistical difference. All statistical analyses were performed with R (http://www.R-project.org) and Empowerstats software (www.empowerstats.com, X& Y solutions, Inc.).

**Table 1:** Baseline characteristics of participants (*N* = 200).

**NEC**	**Case (*n* = 93)**	**Control (*n* = 107)**	**Standardized difference**	***p*-value**
Gestational age (weeks, mean ± SD)	32.74 ± 2.52	32.46 ± 2.75	0.10 (−0.17, 0.38)	0.466
Birth weight (g, mean ± SD)	1,733.83 ± 504.75	1,793.36 ± 581.09	0.11 (−0.17, 0.39)	0.444
Age [h, M(Q1–Q3)]	0.42 (0.35–0.87)	0.48 (0.36–0.94)	0.00 (−0.28, 0.28)	0.993
Sex (*n*, %)			0.18 (−0.10, 0.46)	0.209
Male	43 (46.24%)	59 (55.14%)		
Female	50 (53.76%)	48 (44.86%)		
PLR (mean ± SD)	76.21 ± 34.64	61.19 ± 19.36	0.54 (0.25, 0.82)	<0.001
CRP [mg/L, M(Q1–Q3)]	4.89 (1.26–18.00)	3.30 (2.33–6.13)	0.48 (0.20, 0.76)	<0.001
VD (*n*, %)			0.08 (−0.20, 0.36)	0.577
No	71 (76.34%)	78 (72.90%)		
Yes	22 (23.66%)	29 (27.10%)		
CA (*n*, %)			0.02 (−0.26, 0.29)	0.907
No	54 (58.06%)	63 (58.88%)		
Yes	39 (41.94%)	44 (41.12%)		
PE (*n*, %)			0.38 (0.10, 0.66)	0.008
No	40 (43.01%)	66 (61.68%)		
Yes	53 (56.99%)	41 (38.32%)		
GDM (*n*, %)			0.27 (−0.01, 0.55)	0.061
No	46 (49.46%)	67 (62.62%)		
Yes	47 (50.54%)	40 (37.38%)		

*NEC, necrotizing enterocolitis; PLR, platelet-to-lymphocyte ratio; CRP, C-reactive protein; VD, vaginal delivery; CA, chorioamnionitis; PE, preeclampsia; GDM, gestational diabetes mellitus*.

**Table 2:** Univariate analysis for NEC.

	**Statistics**	**OR (95% CI)**	***p-*Value**
PLR	68.18 ± 28.48	1.02 (1.01–1.04)	0.0005
PLR tripartite group			
Low	67 (33.50%)	1.0	
Middle	66 (33.00%)	0.84 (0.41–1.71)	0.6313
High	67 (33.50%)	3.68 (1.80–7.52)	0.0004
PLR threshold value group			
<40	22 (11.00%)	1.0	
≥40, <80	133 (66.50%)	0.77 (0.31–1.91)	0.5736
≥80, <100	28 (14.00%)	1.60 (0.52–4.93)	0.4127
≥100	17 (8.50%)	9.00 (1.65–49.14)	0.0112
Gestational age (weeks, mean ± SD)	32.59 ± 2.64	1.04 (0.94–1.16)	0.4643
Birth weight (g, mean ± SD)	1,765.68 ± 546.38	1.00 (1.00–1.00)	0.4416
CRP (*n*, %)	8.86 ± 13.72	1.05 (1.02–1.08)	0.0020
CA (*n*, %)			
No	117 (58.50%)	1.0	
Yes	83 (41.50%)	1.03 (0.59–1.82)	0.9072
PE (*n*, %)			
No	106 (53.00%)	1.0	
Yes	94 (47.00%)	2.13 (1.21–3.76)	0.0087
GDM (*n*, %)			
No	113 (56.50%)	1.0	
Yes	87 (43.50%)	1.71 (0.97–3.01)	0.0621

*NEC, necrotizing enterocolitis; CI, confidence interval; OR, odds ratio; PLR, platelet-to-lymphocyte ratio; CRP, C-reactive protein; VD, vaginal delivery; CA, chorioamnionitis; PE, preeclampsia; GDM, gestational diabetes mellitus*.

**Table 3:** Relationship between PLR and NEC in different models.

**Variable**	**Crude Model**		**Model I**		**Model II**	
	**OR (95% CI)**	***p*-value**	**OR (95% CI)**	***p*-value**	**OR (95% CI)**	***p*-value**
**PLR value group**						
<40	1.0		1.0		1.0	
≥40, <80	0.77 (0.31–1.91)	0.5736	0.78 (0.31–1.99)	0.6079	1.08 (0.37–3.13)	0.8871
≥80, <100	1.60 (0.52–4.93)	0.4127	1.57 (0.49–4.96)	0.4454	2.58 (0.70–9.47)	0.1542
≥100	9.00 (1.65–49.14)	0.0112	9.93 (1.75–56.25)	0.0095	18.82 (2.93–120.98)	0.0020

*Crude model adjusted for: None. Model I adjusted for gestational age, birth weight, sex, and VD. Model II adjusted for gestational age, birth weight, sex, VD, CA, PE, GDM, and CRP. PLR, platelet-to-lymphocyte ratio; NEC, necrotizing enterocolitis; OR, odds ratio; CI, confidence interval; VD, vaginal delivery; CA, chorioamnionitis; PE, preeclampsia; GDM, gestational diabetes mellitus; CRP, C-reactive protein*.

## Results

### Patient Characteristics

There was no significant difference in patient characteristics with respect to gestational age, birth weight, sex, rate of vaginal delivery (VD), rate of chorioamnionitis, and gestational diabetes mellitus between the two groups (all *p* > 0.05). Meanwhile, the NEC group had significantly higher PLR levels (76.21 ± 34.64 vs. 61.19 ± 19.36, *p* < 0.001), C-reactive protein (CRP) levels [4.89 (1.26–18.00) vs. 3.30 (2.33–6.13), *p* < 0.001], and rate of mothers with pre-eclampsia than the control group (Table 1).

### Risk Factors

On univariate analysis, the risk factors for NEC were PLR [odds ratio (OR): 1.02 (95% confidence interval (CI): 1.01–1.04), *p* = 0.0005], CRP [OR: 1.05 (95% CI: 1.02–1.08), *p* = 0.002], and pre-eclampsia [OR: 2.13 (95% CI: 1.21–3.76), *p* = 0.0087] (Table 2).

On multivariate analysis, only PLR > 100 within 1 week before NEC diagnosis was an independent risk factor for NEC [OR: 9.00 (95% CI: 1.65–49.14), *p* = 0.0112]. There was a positive association between PLR and preterm NEC (Table 3). Generalized additive model curve analysis to evaluate the diagnostic capability of PLR for NEC (Figure 2) showed that PLR > 100 is the optimal predictive cutoff value. In model II, PLR > 100 was closely associated with NEC [OR: 18.82 (95% CI: 2.93–120.98), *p* = 0.002] (Table 3).

## Discussion

### Despite the Profound Mortality Risk of Preterm NEC, Reliable Biomarkers for Its Early Diagnosis Are Still Lacking

Necrotizing enterocolitis is one of the main causes of death of premature infants (14). However, the etiology and pathogenesis of NEC remain unclear to date. Some studies have shown that NEC is related to immature intestinal development, ischemia, intestinal flora imbalance, decreased mucin barrier, increased intestinal osmotic pressure, decreased intestinal immunity, and the methods of intestinal feeding. These multifactorial events ultimately lead to oxidative stress, inflammation, and necrosis in the neonatal gut (15–18). Accordingly, the diagnosis of NEC depends on a combination of the clinical symptoms (including vomiting, ventosity, and bloody stool) and radiological features (including gastrointestinal accumulation of gas, portal venous gas, and pneumoperitoneum). The most commonly used clinical grading diagnosis system remains to be the modified Bell's staging criteria. Most cases of NEC are diagnosed in the second or third Bell's stage.

The lack of early diagnostic markers and specific symptoms makes it challenging to distinguish early NEC from sepsis, apnea, and feeding intolerance (19–22). Another challenge is the increasing incidence of non-infectious enteritis [e.g., food protein-induced enterocolitis syndrome, food protein-induced rectal colitis, and food protein-induced enteropathy (23–25)] in recent years, making the selection of appropriate laboratory tests and reasonable antibiotic use difficult. Therefore, exploring the early biomarkers of NEC is particularly important for the early differentiation of NEC from other types of enteritis and to avoid unnecessary antibiotic abuse. However, identifying NEC patients even before making a diagnosis remains a challenge for the neonatologists.

Sepsis influences platelet count (26) as platelet–leukocyte interaction regulates inflammation and hemostasis. Activated platelets can express P-selectin and glycoprotein IIIa on their cell surface. This can in turn interact with the selectin receptor P-selectin glycoprotein ligand-1 (PSGL-1), expressed on leukocytes, and the selectin receptor GpIIb, expressed on neutrophils, to mediate cell extravasation and tissue damage (27–29). An animal study using an infectious mouse model reported that a high PLR is associated with inflammation in the early stages of infection (30). It is inferred that platelet activation and the platelet–leukocyte complex are involved in the early stages of inflammatory response and tissue damage.

Increasing studies have shown that the indicators of platelet count, such as the PLR, can predict acute inflammatory and cardiovascular events, prothrombotic states, and neoplastic and metabolic diseases (31–37). They are also helpful for diagnosing inflammatory rheumatic diseases and predicting inflammation-related morbidities (38). A recent study showed that PLR is positively correlated with early-onset neonatal sepsis (EOS) in preterm neonates, and it can thus be added as an additional diagnostic indicator for EOS (10). Erzat et al. have shown that the risk of preterm premature rupture of the membranes (PPROM) is significantly increased when the PLR is >117.14. A high PLR was found to be independent risk factor of PPROM, mediating adverse maternal and neonatal outcomes (39).

Regarding the association between NEC and PLR, a PLR of >100 within 1 week after clinical abnormalities before NEC diagnosis was found to be a risk factor for NEC. Specifically, there was a positive association between PLR and preterm NEC, with a high PLR of >100 being the optimal cutoff for predicting preterm NEC.

This study has some limitations, including the small sample size and single-center design. The diagnostic grade of NEC is not stratified in great detail. Despite these limitations, our findings may be helpful for lowering unreasonable antibiotic abuse, which can in turn protect the intestinal microbial flora stability in premature infants. Further studies are needed to investigate the relationship between the different levels of NEC and the PLR value and to create NEC predictive models, especially in more preterm neonates.

In conclusion, a PLR > 100 within 1 week after clinical abnormalities is significantly associated with the occurrence of preterm NEC and can thus be used for its early diagnosis. Antibiotic use can be delayed in preterm infants presenting with NEC symptoms (e.g., blood in the stool or abdominal distention) if their PLR is <100. In the future, we will choose antibiotics more carefully while managing NEC according to the ratio of PLR.

## Data Availability Statement

The raw data supporting the conclusions of this article will be made available by the authors, without undue reservation.

## Ethics Statement

The studies involving human participants were reviewed and approved by Medical Ethics Committee of West China Second Hospital of Sichuan University. Written informed consent from the participants' legal guardian/next of kin was not required to participate in this study in accordance with the national legislation and the institutional requirements. Written informed consent was not obtained from the individual(s) for the publication of any potentially identifiable images or data included in this article.

## Author Contributions

JZ contributed to the data analysis and interpretation and prepared the initial draft. HW contributed to the experimental design, data collection, data analysis, result interpretation, and manuscript preparation. All authors contributed to the article and approved the submitted version.

## Funding

This work was supported by Application Foundation Project of Science and Technology Department of Sichuan Province, 2021YJ0171.

## Conflict of Interest

The authors declare that the research was conducted in the absence of any commercial or financial relationships that could be construed as a potential conflict of interest.

## Publisher's Note

All claims expressed in this article are solely those of the authors and do not necessarily represent those of their affiliated organizations, or those of the publisher, the editors and the reviewers. Any product that may be evaluated in this article, or claim that may be made by its manufacturer, is not guaranteed or endorsed by the publisher.
